# Molecular and Histological Evaluation of Sheep Ovarian Tissue Subjected to Lyophilization

**DOI:** 10.3390/ani11123407

**Published:** 2021-11-29

**Authors:** Daniela Bebbere, Amir Arav, Stefano Mario Nieddu, Giovanni Pietro Burrai, Sara Succu, Pasquale Patrizio, Sergio Ledda

**Affiliations:** 1 Department of Veterinary Medicine, University of Sassari, 07100 Sassari, Italy; nieddino@hotmail.it (S.M.N.); gburrai@uniss.it (G.P.B.); succus@uniss.it (S.S.); giodi@uniss.it (S.L.); 2 FertileSAFE Ltd., 11 Haharash St, Ness Ziona 7403118, Israel; fertilesafe@gmail.com (A.A.); pxp612@med.miami.edu (P.P.); 3 Mediterranean Center for Disease Control (MCDC), University of Sassari, 07100 Sassari, Italy; 4 Division Reproductive Endocrinology & Infertility, University of Miami, Miller School of Medicine, Don Soffer Clinical Research Center, 1120 NW 14th Street, Miami, FL 33136, USA

**Keywords:** lyophilization, ovarian tissue, vitrification, rehydration, gene expression, histology

## Abstract

**Simple Summary:**

Freeze-drying (or lyophilization) is a method to preserve cells and tissues in which frozen material is dried by sublimation of ice. One of the main advantages is that nitrogen and dry ice are no longer required for the storage and shipment of biological material, which can be kept at room temperature or 4 °C, resulting in enormous reductions in costs. Although widely used to preserve biomolecules and macromolecular assemblies, freeze-drying of cells and tissues is currently experimental. Here, we lyophilized sheep ovarian tissue with a novel device named Darya and assessed effects on tissue integrity and gene expression. We show that ovarian tissue survives lyophilization procedures, maintaining its general structure and reacting to the different experimental steps by regulation of specific genes. Our results contribute to the optimization of protocols to freeze-dry ovarian tissues and may find application in programs of animal and human reproductive tissue preservation.

**Abstract:**

Cryopreservation is routinely used to preserve cells and tissues; however, long time storage brings many inconveniences including the use of liquid nitrogen. Freeze-drying could enable higher shelf-life stability at ambient temperatures and facilitate transport and storage. Currently, the possibility to freeze-dry reproductive tissues maintaining vitality and functions is still under optimization. Here, we lyophilized sheep ovarian tissue with a novel device named Darya and a new vitrification and drying protocol and assessed effects on tissue integrity and gene expression. The evaluation was performed immediately after lyophilization (Lio), after rehydration (LR0h) or after two hours of in vitro culture (IVC; LR2h). The tissue survived lyophilization procedures and maintained its general structure, including intact follicles at different stages of development, however morphological and cytoplasmic modifications were noticed. Lyophilization, rehydration and further IVC increasingly affected RNA integrity and caused progressive morphological alterations. Nevertheless, analysis of a panel of eight genes showed tissue survival and reaction to the different procedures by regulation of specific gene expression. Results show that sheep ovarian tissue can tolerate the applied vitrification and drying protocol and constitute a valid basis for further improvements of the procedures, with the ultimate goal of optimizing tissue viability after rehydration.

## 1. Introduction

Long-term storage of biological samples in medical applications may be achieved by two different approaches: cryopreservation or lyophilization [1]. Cryopreservation allows preservation of cells, tissues, or any other biological construct by cooling samples to very low temperatures [2]. It has become the most widely used and reliable approach to maintain cell or tissue structure and functional integrity during transportation and storage. Despite being currently considered the gold standard for the storage of active cells, it has some important limitations related to costs, as it requires a continuous and guaranteed supply of liquid nitrogen, regular maintenance, dedicated space, and trained staff; in addition, it greatly complicates sample transport and has a heavy carbon footprint due to the use of liquid nitrogen. Such limitations could be overcome by the use of lyophilization. By drying tissues, samples could be stored and transported at ambient temperatures or at 4 °C. Lyophilization (or freeze-drying or vit-drying) is the process of drying a frozen sample via sublimation of ice (freeze-drying), by transiting ice directly to the gas phase without passing through the intermediate liquid one [3] or by desorption of glass material (vitrified) into gas (Vit-Drying [4]). Lyophilization has a long history of successful use in different areas of medicine and the food industry, however, most applications of freeze-drying or drying of mammalian cells could only preserve the integrity and/or functionality of certain subcellular components [5,6,7,8,9,10,11,12]. For example, freeze-drying of EBV-transformed B-lymphoblastoid cells could stabilize the total RNA in the cells for routine diagnostics [13]. Surface-labeled, freeze-dried lymphocytes can be used as the reference material for counting CD4+ T cells [14,15]. The sperm chromatin structure in freeze-dried spermatozoa is largely intact and can be used to fertilize oocytes by intracytoplasmic sperm injection in a range of species including mouse, rat, cat, dog, rabbit, bull, pig, horse, chimpanzee, giraffe, jaguar, weasel, long-haired rat, and human, with offspring reported in some species [16,17,18,19,20,21,22,23,24,25,26,27,28,29]. Despite being developed many years ago [30], the possibility to freeze-dry cells or tissues maintaining vitality and functions is still under optimization, and this also applies for gonadic tissues. Currently, ovarian tissue is most commonly preserved by cryopreservation. Despite various degrees of success reported in different species, which included morphological, cytological, and molecular alterations observed after cryopreservation with different approaches [31,32,33,34,35,36,37,38], ovarian tissue banking was recently accepted as a fertility-preservation technique by the American Society for Reproductive Medicine and is no longer considered experimental [39]. Conversely, procedures to lyophilize ovarian tissue are still in progress and very limited data were reported to date; to our knowledge, only Lee et al. described successful dry-preservation of cat ovarian tissue, with at least partial follicle survival after rehydration (2019) [40].

The aim of this study was to investigate the histological and molecular structure of sheep ovarian tissue after lyophilization with a new freeze-drying device named Darya (FertileSafe, Nes-Ziona, Israel; [41]) and by using a new method of drying following vitrification (Vit-Drying). These preliminary results will contribute to the improvement of the experimental procedures for the lyophilization of reproductive tissues and will pave the way for application in fertility preservation programs, both in the animal and human fields.

## 2. Materials and Methods

All chemicals in this study were purchased from Sigma-Aldrich S.r.l. (Milan, Italy) unless stated otherwise.

### 2.1. Ethics Approval

The ovaries used for in vitro experiments were collected at a local slaughterhouse in Sardinia, Italy, which does not require ethics approval.

### 2.2. Sample Collection

Ovaries were collected from regularly slaughtered ewes aged between 2 and 4 years. Immediately after collection, they were placed in a balanced saline solution (Dulbecco saline phosphate buffer, PBS) added with penicillin (50 mg/mL) and streptomycin (50 mg/mL) and maintained at the constant temperature of 27–30 °C. Ovaries were brought to the laboratory within 2 h of recovery and placed in a glass plate to be processed. They were washed twice in PBS and then placed in Dissection Medium (DM) [TCM199 with 25 mM N2 HydroxyethylpiperazineN2 ethan sulfonic Acid (HEPES) to stabilize pH, 50 IU/mL streptomycin, 50 IU/mL penicillin, and 0.005 M NaHCO3 and polyvinyl alcohol (PVA) 0.1% (*w*/*v*)] at pH 7.22 ± 0.1 and 4 °C. Ovaries were sectioned with a sterile microblade and tissue sections of 1 mm^3^ were collected.

### 2.3. Experimental Design

**Figure 1:**
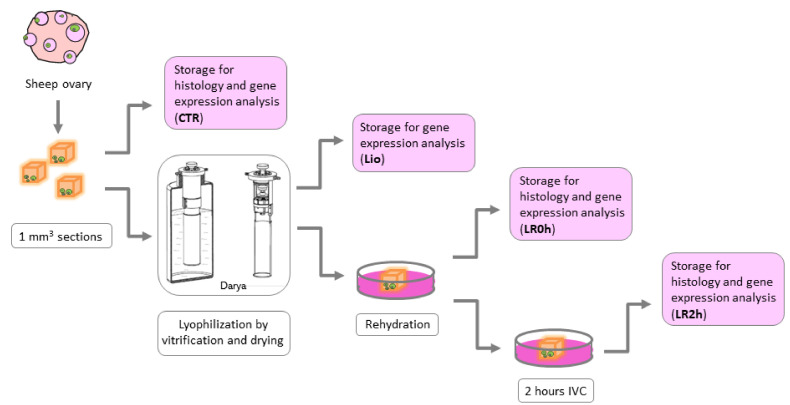
Description of the experimental design.

The sections of ovarian tissue (1 mm^3^) were immediately subjected to vitrification and drying. After rehydration, tissue sections were cultured in vitro for two hours. Sections of fresh ovarian tissue were included as controls (CTR; Figure 1). Eight animals were used for this study; each experimental group included sections derived from the ovaries of each ewe (eight biological replicates per group), as follows:

- CTR (*n* = 8): Sections of fresh ovarian tissue stored for further analysis immediately after dissection.
- Lio (*n* = 8): Sections of ovarian tissue stored for further analysis immediately after lyophilization by vitrification procedure (without warming procedure).
- LR0h (*n* = 8): Sections of ovarian tissue stored for further analysis after lyophilization and rehydration procedures.
- LR2h (*n* = 8): Sections of ovarian tissue stored for further analysis after lyophilization and rehydration procedures followed by 2 h of in vitro culture.

Tissue integrity was assessed by histological analysis after lyophilization procedures (LR0h and LR2h) and in fresh controls (CTR). All experimental groups were subjected to analysis of expression of a panel of eight genes.

### 2.4. Tissue Vitrification and Drying

Tissue sections were exposed to two different solutions (equilibration and vitrification solutions). First, samples were immersed for 25 min in 1 mL of equilibration solution (ES) consisting of TCM-199, 25 mM HEPES, 7.5% ethylene glycol (EG), 7.5% dimethyl sulfoxide (DMSO), and 20% fetal calf serum (FCS). Subsequently, samples were transferred into 1 mL of vitrification solution [VS: TCM-199 with HEPES 25 mM, EG 18%, DMSO 18%, bovine serum albumin (BSA) 0.6%, and trehalose 0.5 M] for 15 min [42]. 

Samples were immediately subjected to Vit-dry procedures using Darya lyophilizer device (FertileSafe, Nes-Ziona, Israel), which allows to control the condensation temperature and reaches a vacuum pressure of 80 mTorr in less than 10 s. Before use, the Darya device was sterilized in an autoclave.

Tissue sections were lyophilized at −50 °C for 20 h, then at −35 °C for two hours, at −25 °C for a further two hours, and, finally, they were directly plunged into liquid nitrogen.

### 2.5. Rehydration

Rehydration was achieved simultaneously with temperature recovery by sequential exposure of the samples to TCM-199 solutions with 20% FCS and decreasing sucrose concentrations (1 M, 0.5 M and 0.25 M) at 38.6 °C. Samples were kept inside each solution for 5 min. Tissue samples for gene expression analysis were immediately stored in RNALater™ (Qiagen, Hilden, Germany) or transferred to a Petri dish containing culture medium (IVC: TCM-199 with 100 μM cysteamine, 10% FCS, 2.1 g/L sodium bicarbonate, 0.36 mM pyruvate) and stored in RNALater™ after 2 h of in vitro culture. Samples were stored at −80 °C until further processing. Tissue samples for histological analysis were immediately stored in Bouin’s solution.

### 2.6. Gene Expression Analysis

Gene expression analysis by real-time PCR was performed and is described according to MIQE guidelines [43] and in line with recent recommendations [44].

### 2.7. Total RNA Isolation and Reverse Transcription

Tissue samples for molecular analysis were plunged into RNALater (ThermoFisher Scientific) immediately after each treatment and stored at −80 °C until RNA isolation. Total RNA was isolated using 1 mL TRIzol reagent (Invitrogen Corporation, Carlsbad, CA, USA) and treated with DNase I (Invitrogen Corporation) according to manufacturer’s protocols. The resulting RNA quantity and purity were spectroscopically checked with NanoDropLite (Fisher Scientific S.A.S., Illkirch Cedex, France), while RNA integrity was evaluated by electrophoresis in a 1% agarose gel in Tris Borate EDTA Buffer (1 µg RNA per sample). Five hundred ng total RNA from each sample were reverse transcribed in a 20 μL reaction with 50 mM Tris HCl (pH 8.3), 75 mM KCl, 3 mM MgCl2, 5 mM DTT, 1 mM dNTPs, 2.5 μM Random Hexamer primers, 20 U of RNaseOUT™, and 100 U of SuperScript™ III RT (all provided by Invitrogen Corporation). Negative control reactions (without the enzyme) were carried out to confirm the absence of genomic DNA contamination. The reaction tubes were incubated at 25 °C for 10 min, at 42 °C for 1 h, and, finally, at 70 °C for 15 min to inactivate the reaction.

### 2.8. Real Time Polymerase Chain Reaction

Relative quantification of transcripts was performed by real-time polymerase chain reaction (PCR) in a Rotor-Gene Q 5 plex HRM (Qiagen). The PCR was performed in a 15 μL reaction volume containing 7.5 μL 2× Qiagen PCR Master Mix (Qiagen), 200 nM of each primer (Table 1) and cDNA equivalent to ~20 ng RNA. The PCR protocol consisted of two incubation steps (50 °C for 5 min and 95 °C for 2 min), followed by 40 cycles of amplification program (95 °C for 15 s, gene-specific annealing temperature for 30 s, Table 1), a melting curve program (65–95 °C, starting fluorescence acquisition at 65 °C and taking measurements at 10 s intervals until the temperature reached 95 °C) and finally a cooling step to 4 °C. Fluorescence data were acquired during the elongation step. To minimize handling variation, all samples were analyzed within the same run using a PCR master mix containing all reaction components apart from the sample. The PCR products were analyzed by generating a melting curve to check the specificity and identity of the amplification product. For each primer pair, the efficiency of the PCR reaction was determined by building a standard curve with serial dilutions of a known amount of template, covering at least three orders of magnitude, so that the calibration curve’s linear interval included the interval above and below the abundance of the targets. Only primers achieving an efficiency of reaction between 90 and 110% (3.6 > slope > 3.1) and a coefficient of determination r^2^ > 0.99 were used for the analysis.

Target gene expression was normalized against the geometrical mean of three housekeeping gene expressions: ribosomal protein L19 (*RPL19*), actin B (*ACTB*) and succinate dehydrogenase complex flavoprotein, subunit A (*SDHA*).

### 2.9. Histology

Samples of LR0h, LR2h, and CTR experimental groups were fixed in Bouin’s solution for 12 h, paraffin-embedded, 3 µm sectioned, stained in Hematoxylin Eosin (HE) and Masson’s Trichrome, and visualized under an optical microscope. Cortical and medullary tissues were histologically evaluated to identify potential morphological changes affecting follicular structures and the surrounding parenchyma. Digital computer images were recorded with a Nikon (Tokyo, Japan) Ds-fi1 camera.

### 2.10. Statistical Analysis

Data were analyzed with GraphPad Prism version 8.0.0 for Windows, GraphPad Software, San Diego, CA, USA. After testing for normality using a Kolmogorov–Smirnov test, gene expression data were analyzed with the General Linear Model analysis of variance (ANOVA), followed by Tukey’s post-hoc comparison (if ANOVA was significant). Differences were considered significant when *p* < 0.05.

## 3. Results

### 3.1. RNA Integrity

**Figure 2:**
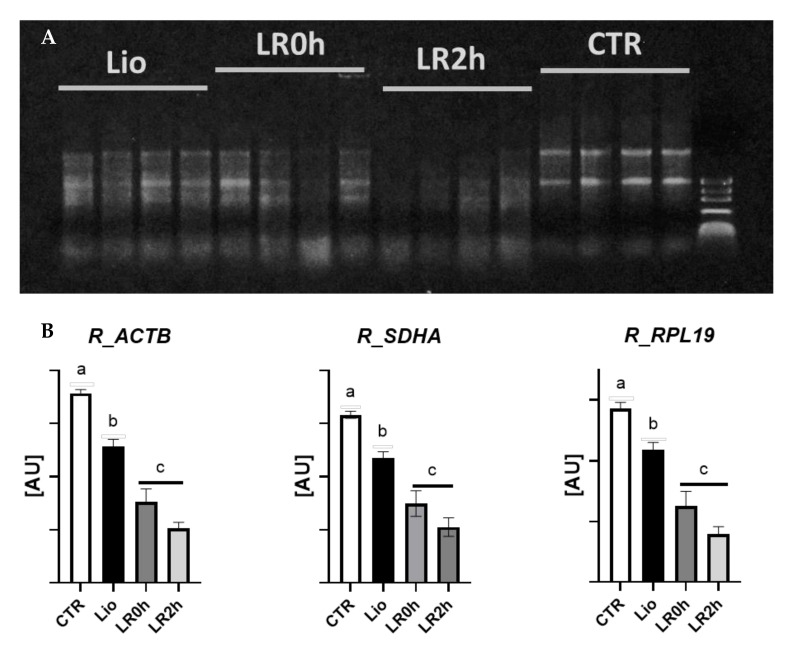
(**A**). Evaluation of total RNA integrity (1 µg per sample) by electrophoretic separation on 1% agarose gel in TBE buffer 0.5 X. (**B**). Relative expression of *ACTB, SDHA*, and *RPL19* in ovarian tissue subjected to lyophilization (Lio), lyophilization and rehydration (LR0h), lyophilization, rehydration and two-hour in vitro culture (LR2h), and in fresh tissue (CTR). Transcript abundance is expressed in arbitrary units ([AU] mean values ± SEM of 8 samples). Different letters above columns indicate a significant difference between groups (ANOVA *p* < 0.05).

Agarose gel electrophoresis highlighted differences in the integrity of the total RNA isolated from tissues subjected to different treatments (Figure 2A).

Total RNA includes different types of RNA, and ribosomal RNA (rRNA) is quantitatively preponderant (about 80%). For this reason, rRNA subunits (28 S, 18 S, and 5.8 S) may be visualized after electrophoretic separation in agar gel. Visualization of two sharp and clear bands, representing the 28 S and 18 S rRNAs, indicate good RNA integrity. Here, electrophoresis showed excellent integrity for CTR samples; the tissues subjected to the lyophilization process only (Lio) maintained fair integrity (it is possible to distinguish the rRNA bands, but there is a slight smear representing partially fragmented RNA). Tissues subjected to lyophilization and rehydration (LR0h) showed increasing degrees of fragmentation and variability between samples, which further increased after two-hour IVC (LR2h; Figure 2A).

### 3.2. Gene Expression

*ACTB, RPL19*, and *SDHA* are endogenous reference genes suitable for expression studies in different tissues [42,45,46]. In the present experiment, their expression is not stable across experimental groups but reflects the degree of RNA integrity (Figure 2B).

As RNA fragmentation may affect the quantification of gene expression, we compared the expression of target genes before and after normalization against reference gene expression to dissect the variation due to RNA integrity from the variation due to specific gene regulation.

**Figure 3:**
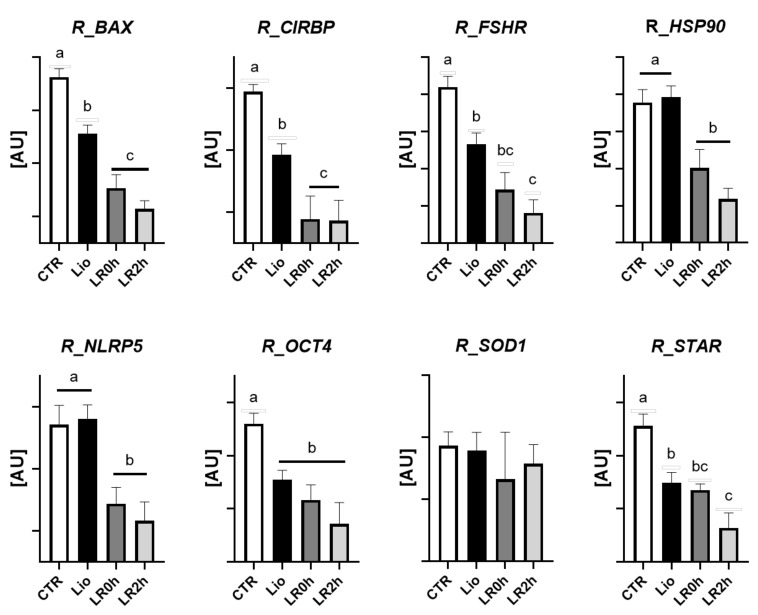
Relative expression of *BAX, CIRBP, FSHR, HSP90b, NLRP5, OCT4, SOD1*, and *STAR* in ovarian tissue subjected to lyophilization (Lio), lyophilization and rehydration (LR0h), lyophilization, rehydration and two-hour in vitro culture (LR2h), and in fresh tissue (CTR). Transcript abundance is expressed in arbitrary units ([AU] mean values ± SEM of 8 samples). Different letters above columns indicate a significant difference between groups (ANOVA *p* < 0.05).

In absence of normalization against the reference genes, five of the eight analyzed genes (*BAX, CIRBP, OCT4, FSHR*, and *STAR*) show patterns of expression similar to the reference genes and in accordance with RNA integrity (Figure 3): the relative quantification of these transcripts show significantly lower levels in tissues subjected to lyophilization (Lio) and rehydration (LR0h and LR2h). The expression of NLRP5 and HSP90b in the lyophilized samples (Lio) is similar to controls, while a decrease is observed after rehydration and IVC (LR0h and LR2h). Finally, SOD1 transcript abundance shows no variation across the four experimental groups.

**Figure 4:**
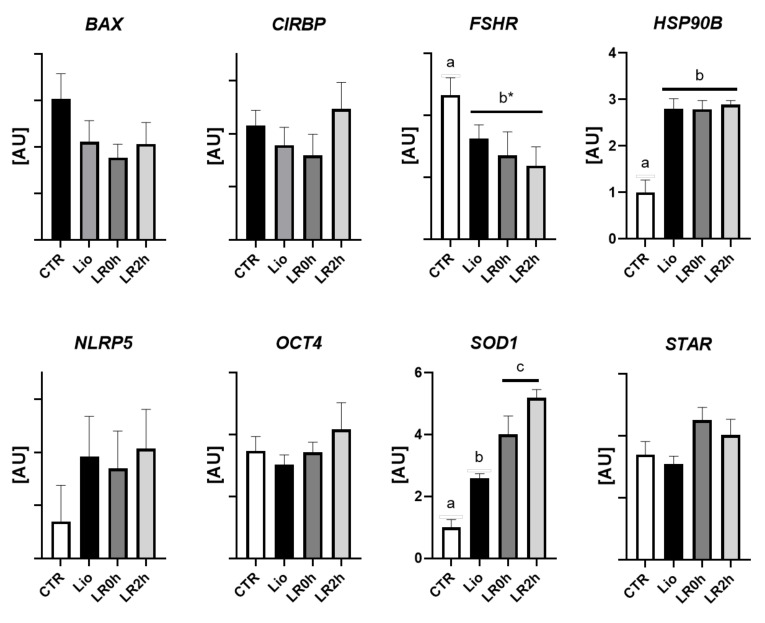
Relative expression of *BAX, CIRBP, FSHR, HSP90b, NLRP5, OCT4, SOD1*, and *STAR* in ovarian tissue subjected to lyophilization (Lio), lyophilization and rehydration (LR0h), lyophilization, rehydration and two-hour in vitro culture (LR2h), and in fresh tissue (CTR) after normalization against the mean expression of three reference genes (*ACTB, RPL19, SDHA*). Transcript abundance is expressed in arbitrary units ([AU] mean values ± SEM of 8 samples). Different letters above columns indicate a significant difference between groups (ANOVA *p* < 0.05); * *p* = 0.056.

Normalization of the target gene expression against the reference gene abundance identified which gene was specifically regulated by the treatment in the live/surviving cells. The abundance of *BAX, CIRPB, NLRP5, OCT4*, and *STAR* mRNAs did not differ following treatments. On the contrary, *HSP90b* expression significantly increased after lyophilization (Lio) and remained stable after rehydration and two-hour IVC (LR0h and LR2h). The expression of *SOD1* increased after lyophilization (Lio) and after dehydration (LR0h) and maintained the high level after IVC (LR2h). Conversely, *FSHR* expression showed a decrease approaching significance (*p* = 0.056; Figure 4) after lyophilization, which was maintained after rehydration and IVC.

### 3.3. Histology

The histological analysis of CTR, LR0h, and LR2h samples showed progressive morphological alterations both in the follicular and in the interstitial and stromal components.

**Figure 5:**
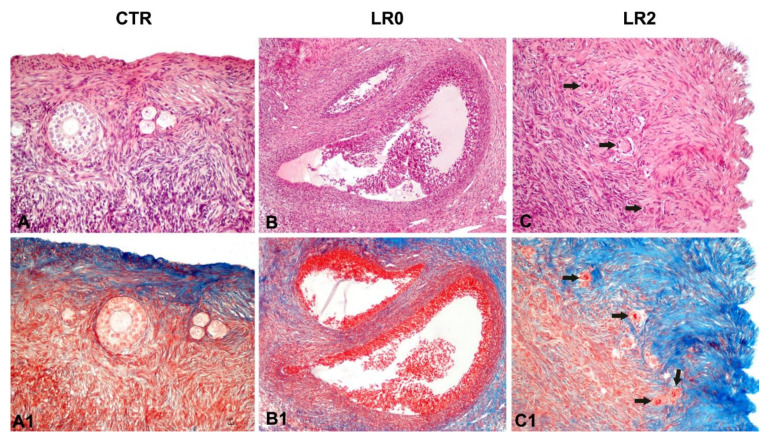
Histological images of CTR (**A**,**A1**), LR0h (**B**,**B1**), and LR2h (**C**,**C1**). Progressive and severe detachment of the granulosa cells from the basement membrane is observed both in LR0h and LR2h samples as well as eosinophilic irregular cytoplasmic vacuolization and severe fragmentation and condensation of the nuclear chromatin (**C**,**C1** arrow). (**A**–**C**): Hematoxylin and eosin; (**A1**–**C1**) Masson’s Trichrome.

Approximately 80% of primordial and primary and 90 to 100% of secondary follicles were characterized by a multifocal detachment of the granulosa cells from the basement membrane (Figure 5B,B1). Multifocal to coalescing scant eosinophilic irregular cytoplasmic vacuolization and severe fragmentation and condensation of the nuclear chromatin (Figure 5C1), with multifocal intranuclear slightly basophilic vacuolization, were noticed. Diffusely, the medullary and cortical stroma were expanded by weakly basophilic edema.

## 4. Discussion

Lyophilization of reproductive tissues is currently experimental. In the context of reproduction, the use of freeze-drying has been successfully applied to mouse spermatozoa, which maintain chromosomal stability [11] and the ability to fertilize oocytes and support normal embryonic development [9]. Conversely, the lyophilization of gonadal tissue, both in male and female, is still being tested. Nevertheless, the many advantages of such cost-effective preservation methods encourage further efforts to optimize protocols and improve the quality of the tissues subjected to such procedure.

The present work showed that sheep ovarian tissue subjected to vitrification/lyophilization procedures survives and reacts by regulating the expression of specific genes.

Moreover, histological analysis indicated structural alterations due to lyophilization, such as the detachment of the granulosa cells from the basal membrane, cytoplasmic modifications, and disaggregation of the nuclear chromatin. However, histologically, the tissue retained its general microscopic features allowing the identification of the main cell types including intact primordial follicles (Figure 5). Nevertheless, additional studies on a higher number of samples at different time points of lyophilization are ongoing to fully characterize and quantify the modification observed in the different stages of follicle development and on the ovarian tissue.

Moreover, histological analysis showed that the tissue maintains its general structure, including intact follicles at different stage of development (Figure 5), with only limited morphological and cytoplasmic modifications.

To achieve ovarian tissue lyophilization, we employed a new benchtop device called “Darya”, recently designed to lyophilize cells and tissues, and previously applied to freeze-dry ram sperm [41]. Freeze drying requires the optimization of three main procedures: freezing, drying, and rehydration. Excessive dehydration can cause macromolecular denaturation and reduction in cell volume, with possible irreversible collapse of the cell membrane [47]. A further deleterious effect is the mechanical stress caused by ice formation around the cells, which forces the cells into the extremely limited space of the unfrozen solution [48]. The Darya instrument allows rapid sublimation at low temperatures below zero which, combined with the action of sugar-based cryoprotective solutions such as Trehalose and DMSO, causes reduced osmotic effects and facilitates sample dehydration.

At present, tissue degeneration after freeze-dry procedures is non-negligible. A deeper understanding of how cells react to the process, together with the specific molecular mechanisms, is beneficial to improve freeze-dry experimental protocols. In our experimental design, we have attempted to understand the variation in gene expression after lyophilization procedure, after lyophilization and rehydration, and during a 2 h post-rehydration period of in vitro culture, to dissect the molecular responses specific to each experimental step. The selected gene panel comprises germ-cell specific markers (*NLRP5, POU5F1* [*OCT4*]), genes specifically expressed in supporting somatic cells (*FSHR* and *STAR*), and genes involved in cell stress response (*BAX*, *SOD1*, *CIRBP*, and *HSP90b*). *OCT4* (*POU5F1*) encodes a transcription factor specific of germ cells [49], while *NLRP5* is a maternal effect gene exclusively expressed in oocytes and early embryos [50]; the receptor of the follicle stimulating hormone (*FSHR*) and the steroidogenic acute regulatory protein (*STAR*) are expressed only in granulosa cells; finally, *BAX*, *SOD1*, *CIRBP*, and *HSP90b* encode molecules involved in the cellular response to different stress conditions [51,52,53,54].

The first observation derived from the molecular analysis is that lyophilization procedures cause a partial RNA degradation; dissection of the different experimental steps showed that samples subjected only to lyophilization (Lio) maintained RNA integrity, while after rehydration and subsequent in vitro culture RNA showed significant signs of degradation (Figure 2). These observations suggest that part of the cells did not survive the process and highlight the need of an optimization of these experimental phases to better preserve tissue integrity. In accordance, the analysis of the single genes confirmed a decrease in transcript abundance during the entire lyophilization process, which reflects the decrease in total RNA content (Figure 3).

RNA degradation can compromise a proper gene expression analysis. The presence of damaged RNA molecules can prevent adequate quantification, altering primer binding, enzyme activity (reverse transcriptase and DNA polymerase) and molecule elongation during cDNA synthesis or during PCR. These phenomena can cause an imprecise quantification of both target and reference genes. In the present work, the degradation of RNA clearly affected the transcript levels of all genes expressed in the ovarian tissue. To overcome this criticism and carry out an appropriate evaluation, we examined the expression of the target genes with and without normalization against reference genes (Figure 3 and Figure 4). This allowed us to distinguish the transcript variations due to the overall RNA degradation from the variations due to activation or suppression of specific genes.

Comparison of the two sets of expression indeed identified a specific response to lyophilization in terms of gene regulation, which indicates that surviving cells precisely up- or down-regulated certain genes following lyophilization, rehydration, or subsequent in vitro culture.

The expression of all genes was detected in all experimental groups, indicating the survival of both germ and somatic cells to the entire experimental process. The relative quantification of the reference genes (*ACTB*, *RPL19*, and *SDHA*) reflects the levels of RNA degradation, with a significant decrease between controls and lyophilized samples, and a further decrease following rehydration (Figure 3); such patterns indicate a negative effect of lyophilization and rehydration, which possibly cause death of part of the cells, whose RNA undergoes degradation.

The analysis of the target genes in absence of normalization showed patterns in accordance with RNA degradation in four cases: *BAX, CIRBP, FSHR*, and *STAR*. *OCT4* showed a significant decrease in all treated groups, while *NLRP5* and *HSP90b* showed similar levels of expression between controls and lyophilization (Lio) and a significant decrease following rehydration. On the contrary, *SOD1* did not show variations in expression (Figure 3).

To identify the genes that responded specifically to lyophilization in terms of up- or down-regulation, we then compared the expression levels after normalization against the reference genes. Five genes showed similar transcript levels among the four experimental groups: *BAX*, *CIRBP*, *NLRP5*, *OCT4*, and *STAR*, while *FSHR* showed a difference close to significance between controls and the three treated groups (Figure 4). These results indicate that the surviving tissue maintained proper function, both in somatic cells (*FSHR* and *STAR*), and germlines (*NLRP5* and *OCT4*). The expression patterns of *HSP90b* and *SOD1* clearly show that the tissue that survived the conservation process activated the expression of genes involved in the cellular response to stress conditions (Figure 4). *HSP90b* mRNA levels increased after lyophilization (Lio) and maintained similar levels following rehydration and subsequent IVC (L0R and L2R). HSP90b is involved in the correct folding of proteins that have become structurally unstable due to exposure to various types of cell stress (heat or cold shock, hyperosmotic stress, or heavy metal toxicity) [51,52,53,54]. Its steady upregulation indicates that exposure to vit-dry procedures exerts negative effects on protein stability. Conversely, *SOD1* showed two significant increases following lyophilization (Lio) and rehydration (L0R and L2R; Figure 5). The enzyme superoxide dismutase 1, encoded by *SOD1*, is responsible for the elimination of free radicals (Reacting Oxygen Species [ROS]) achieved by converting superoxide ions into molecular oxygen and hydrogen peroxide; the increasing *SOD1* expression we observed following lyophilization, rehydration and IVC is, therefore, an index of growing oxidative stress (Figure 4).

The results of histological analysis indicate structural alterations due to lyophilization, such as the detachment of the granulosa cells from the basal membrane, cytoplasmic modifications and disaggregation of the nuclear chromatin. However, the tissue retained its general structure, and it is possible to distinguish morphologically intact primordial follicles (Figure 5). These histological observations, together with the results of the molecular analysis, suggest that at least part of the tissue was preserved and able to tolerate the lyophilization protocol applied in the present study.

Our observations are in partial agreement with the study performed on cat ovarian tissue by Lee et al. [40], who characterized the influence of microwave-assisted dehydration after exposure to trehalose on morphology and viability of living ovarian tissues. While their preservation procedure did not involve cryopreservation, the two protocols share the dehydration and rehydration steps, which may irreparably damage the vital functions of the tissue. Whereas we observed structural alterations, they report proper morphology and DNA integrity of follicles and stromal cells, together with a partially maintained transcriptional activity following 10 min of drying.

Despite the reported molecular and structural alterations, these studies on dry-preservation techniques are encouraging and provide foundation for protocol optimization. The abundant literature on ovarian tissue cryopreservation (both slow freezing and vitrification) also describes alteration of tissue integrity in terms of follicular and stromal morphology, cell viability, DNA integrity, and gene expression [31,32,33,34,35,36,37,38]. Nevertheless, extensive research in different species has led to optimization of the techniques and significant improvement of tissue integrity following cryopreservation, with its recent inclusion in clinical practice of fertility preservation [39].

## 5. Conclusions

In conclusion, the present study provided a preliminary microscopic and molecular evaluation of the effects of lyophilization on ovine ovarian tissue. The analysis of the different steps dissected the specific effects of lyophilization or rehydration of the sample. The results indicate that at least part of the ovarian tissue tolerated the vit-dry preservation protocol and may be useful for further improvements of the procedures, with the ultimate goal of optimizing tissue viability after rehydration.

## Figures and Tables

**Table 1:** Primers used for gene expression analysis by Real Time PCR.

Gene	Symbol	Sequence	Accession Number	Annealing Temp.	Size (bps)
Actin β	*ACTB*	F: 5′ ttcctgggtatggatcctg 3′ R: 5′ ggtgatctccttctgcatcc 3′	NM_001009784	60 °C	162
BCL2 associated X Protein	*BAX*	F: 5′ ctccccgagaggtctttttc R: 5′ tcgaaggaagtccaatgtcc	XM_004015363	58 °C	176
Cold inducible RNA binding protein	*CIRBP*	F: 5′ gagggctgagttttgacacc 3′ R: 5′ atgggaagtctgtggatggg	XM_004008776	60 °C	190
Follicle stimulating hormone receptor	*FSHR*	F: 5′ agtcttcctctgccaggaca 3′ R: 5′ cttctgggatgactcgaagc 3′	NM_001009289	60 °C	107
Heat shock protein 90 b	*HSP90B*	F: 5′ tggagatcaaccctgacca R: 5′ gggatcctcaagcgagaag	XM_004018854	58 °C	143
NLR family pyrin domain containing 5	*NLRP5*	F: 5′ cagcctccaggagttctttg 3′ R:5′ gacagcctaggagggtttcc 3′	XM_027978862	59 °C	212
POU domain, class 5, transcription factor 1	*POU5F1* *(OCT4)*	F: 5′ gaggagtcccaggacatcaa 3′ R:5′ ccgcagcttacacatgttct 3′	XM_012101009	56 °C	204
Ribosomal protein L9	*RLP19*	F: 5′caactcccgccagcagat 3′ R:5′ ccgggaatggacagtcaca 3′	XM_004012836	56 °C	127
Steroidogenic acute regulatory protein	*STAR*	5′ cccatggagaggctttatga 3′ 5′ cagccaactcgtgagtgatg 3′	NM_001009243	60 °C	130
Succinate Dehydrogenase	*SDHA*	F: 5′catccactacatgacggagca 3′ R:5′ atcttgccatcttcagttctgcta 3′	XM_012125144	60 °C	90
Superoxide dismutase 1	*SOD1*	F: 5′ ggcaatgtgaaggctgacaa 3′ R:5′ aagaccagatgacttgggca 3′	NM_001145185	58 °C	130

## Data Availability

The data produced during the current study are available from the corresponding author on reasonable request.
